# Supplementary material for: Reconciling Mining with the Conservation of Cave Biodiversity: A Quantitative Baseline to Help Establish Conservation Priorities
Source: PLoS One. 2016 Dec 20;11(12):e0168348. doi: 10.1371/journal.pone.0168348 (PMC5173368; doi:10.1371/journal.pone.0168348)
Supplement: S1 Dataset — (ZIP) [file pone.0168348.s002.zip › Taxa/Serra Norte/SN_2013/N4E/Lista N4E-75.pdf]

CAVIDADE N4E-0075

Classe	Ordem	Fam/Outros	Gên/Outros	Espécie	S-U	U-S	Zon.
Annelida	Oligochaeta			jovem	1	3	E/P
Arachnida	Acari	(Oribatida)		sp.2	X		P
Arachnida	Acari	(Parasitengonina)		sp.11		X	P
Arachnida	Acari	(Parasitengonina)		sp.16		X	P
Arachnida	Acari	Anystidae	*Erythracarus*	sp.1	X	3	P
Arachnida	Acari	Ixodidae		jovem	X		E
Arachnida	Acari	Rhagidiidae		sp.2		X	P
Arachnida	Amblypygi	Charinidae	*Charinus*	sp.1	1	1	E/P
Arachnida	Amblypygi	Phrynidae	*Heterophrynus*	*longicornis*	1	1	E/P
Arachnida	Araneae	Ctenidae		jovem	1		
Arachnida	Araneae	Drymusidae		jovem	X	X	E
Arachnida	Araneae	Ochyroceratidae		jovem		X	A
Arachnida	Araneae	Oonopidae		jovem		X	E
Arachnida	Araneae	Pholcidae		jovem	X		E
Arachnida	Araneae	Salticidae		jovem	X		E
Arachnida	Araneae	Scytodidae	*Scytodes*	jovem	1		E
Arachnida	Araneae	Segestriidae		jovem	X		E
Arachnida	Araneae	Theridiidae	*Theridion*	sp.4	X	X	P
Arachnida	Araneae	Theridiosomatidae	*Plato*	sp.1	X	X	E/P
Arachnida	Araneae	Uloboridae	*Uloborus*	sp.2	X		E
Arachnida	Opiliones	Escadabiidae		sp.2	X		P
Arachnida	Opiliones	Sclerosomatidae	*Prionostema*	sp.1	1	1	E
Arachnida	Pseudoscorpiones	Bochicidae		sp.1	X		P
Arachnida	Pseudoscorpiones	Chthoniidae	*Pseudochthonius*	sp.1	X		E/P
Chilopoda	Geophilomorpha	Geophilidae		sp.1	1	1	P
Chilopoda	Scolopendromorpha			jovem	1		E
Diplopoda	Glomeridesmida	Glomeridesmidae		sp.2	1		A
Diplopoda	Polydesmida	Fuhrmannodesmidae		sp.1	X		P/A
Diplopoda	Polydesmida	Pyrgodesmidae		sp.1	X	X	P/A
Diplopoda	Spirostreptida	Pseudonannolenidae	*Pseudonannolene*	jovem	1		E
Entognatha	Collembola	Isotomidae		sp.1	X	X	P/A
Entognatha	Collembola	Paronellidae		sp.1		X	E
Entognatha	Collembola	Sminthuroidea		sp.2	X		P
Entognatha	Diplura	Projapygidae		sp.1	X		E
Insecta	Coleoptera	Curculionidae		sp.1	X		E
Insecta	Coleoptera	Hydrophilidae		sp.11	X		E
Insecta	Coleoptera			jovem		X	E
Insecta	Diptera	Cecidomyiidae	Cecidomyiinae	sp.	X	X	E/P
Insecta	Diptera	Limoniidae		sp.		X	E
Insecta	Diptera	Psychodidae	Phlebotominae	sp.	X	X	P/A
Insecta	Diptera	Sciaridae		sp.	X		P
Insecta	Diptera	Tipulidae	Tipulinae	sp.	X	X	E
Insecta	Heteroptera	Cydnidae		jovem		X	P
Insecta	Heteroptera	Veliidae	*Microvelia*	sp.	X	X	E
Insecta	Homoptera	Cixiidae		jovem	X	X	E/P
Insecta	Hymenoptera	Formicidae	*Camponotus*	sp.1	X	X	E/P
Insecta	Hymenoptera	Formicidae	*Paratrechina*	sp.1	X		E
Insecta	Hymenoptera	Formicidae	*Paratrechina*	sp.2	X		E
Insecta	Hymenoptera	Formicidae	*Solenopsis*	sp.1		X	E/P

Insecta	Isoptera	Termitidae	*Diversitermes*	sp.		X	E/P
Insecta	Isoptera	Termitidae	*Nasutitermes*	sp.	X	X	E
Insecta	Lepidoptera	Noctuoidea		sp.5	1		E
Insecta	Lepidoptera	Noctuoidea		sp.7		1	E
Insecta	Orthoptera	Phalangopsidae	*Phalangopsis*	sp.	28	30	E/P/A
Gastropoda	Pulmonata	Bulimulidae	*Naesiotus*	sp.	X	X	E/P/A
Gastropoda	Pulmonata	Subulinidae		sp.3	X	X	P/A
Gastropoda	Pulmonata	Subulinidae		sp.4	X	X	P/A
Gastropoda	Pulmonata	Subulinidae		sp.5		X	E
Gastropoda	Pulmonata	Systrophiidae	*Happia*	sp.	X	X	P/A
Malacostraca	Isopoda	Armadillidae		sp.1	X		E
Malacostraca	Isopoda	Philosciidae		sp.1	X	X	P
Symphyla		Scutigerellidae		sp.	X	X	E/P
Amphibia	Anura	Bufonidae	*Rhinella*	sp.	1		E
Mammalia	Chiroptera	Emballonuridae	*Peropteryx*	*kappleri*	2	2	E/P
